# Response Surface Methodology Optimization for Analytical Microwave-Assisted Extraction of Resveratrol from Functional Marmalade and Cookies

**DOI:** 10.3390/foods12020233

**Published:** 2023-01-04

**Authors:** Widiastuti Setyaningsih, María del Cisne Guamán-Balcázar, Nurul Mutmainah Diah Oktaviani, Miguel Palma

**Affiliations:** 1 Department of Food and Agricultural Product Technology, Faculty of Agricultural Technology, Gadjah Mada University, Jalan Flora, Bulaksumur, Yogyakarta 55281, Indonesia; 2 Department of Chemistry, Universidad Técnica Particular de Loja, San Cayetano Alto, Loja 1101608, Ecuador; 3 Department of Analytical Chemistry, IVAGRO, Faculty of Sciences, University of Cadiz, Campus de Excelencia Internacional Agroalimentario (CeiA3), Campus del Rio San Pedro, 11510 Puerto Real, Cadiz, Spain

**Keywords:** Box–Behnken design, functional foods, method development, optimization, validation

## Abstract

A novel analytical method based on microwave-assisted extraction has been successfully optimized and validated to determine resveratrol from functional marmalade and cookies. The optimization was performed using a Box–Behnken design with three factors: solvent composition (60−100% and 10−70% methanol in water for marmalade and cookies, respectively), microwave power (250−750 W), and solvent-to-solid ratio (20:5−60:5). The main and quadratic effects of solvent composition significantly contributed to the recovery values (*p* < 0.005) for both kinds of samples. Additionally, the solvent-to-solid ratio and the quadratic effect of microwave power also influenced the resveratrol recovery from functional marmalade. Hence, the optimum condition for resveratrol extraction from marmalade (80% methanol, 500 W, solvent-to-solid ratio 40:5) and cookies (80% methanol, 250 W, solvent-to-solid ratio 20:5) was proposed. The extraction kinetics (5−30 min) was then studied to clarify the complete recovery of resveratrol from the food matrices resulting in 15 min as the optimum extraction time. The developed method showed a satisfactory precision indicated by the coefficient of variation (CV) lower than 5.70% for both repeatability and intermediate precision. To check the matrix effects, the developed MAE procedures were applied to a number of commercial marmalade and cookies. The high-fat and fiber cookies resulted in overestimated values due to the interfering ingredients. As a final point, the methods successfully measured the stability of naturally present resveratrol in grape-derived products while preparing functional marmalade and cookies.

## 1. Introduction

Resveratrol (3,5,4′-trihydroxy-trans-stilbene) is a phenolic compound belonging to the stilbenoid group [1]. This compound can naturally be found in natural sources, including in red grapes and their derived product, such as red wine [2]. It is synthesized in plants to protect against biotic and abiotic stress, such as pathogenic attacks, environmental stress, UV irradiation, and fungal infections [3,4]. Resveratrol is essential for human health in preventing cancer, diabetes, Alzheimer’s, Parkinson’s, and cardiovascular diseases [5]. Hence, resveratrol has been used as an ingredient to develop functional food. 

Referring to the natural source of resveratrol, two types of ready-to-eat functional foods based on red grapes were developed, i.e., marmalade and cookies [6]. Marmalade production is one attempt to preserve fruit, including grapes which are rich in resveratrol [7]. The products are among the popular condiments due to ease of storage and relatively longer shelf-life. Another product of mass consumption around the world is cookies, in which recipes can be varied. Thus, adding a functional compound into these matrices requires a careful inspection of the alteration during the processing. Therefore, analytical methods to quantify the compound greatly interest the nutraceutical industries. 

Extraction is the crucial first step in an analytical procedure to determine the targeted compound in a food sample. It is necessary to separate the compound from interferences presently in the matrix for further identification and quantification [8]. Conventionally, resveratrol can be recovered by maceration and Soxhlet extraction, which consume large amounts of solvents, generating a large number of wastes that are harmful to the environment [9,10]. Additionally, the methods use high temperatures in long extraction times, which could degrade the compounds. Hence, advanced techniques, including microwave-assisted extraction (MAE), have been widely applied as an alternative to extract bioactive compounds from either plants or food products. 

The MAE technique operates by radiating microwave energy that penetrates inside the samples, as a result, generating simultaneous heat that endorses the extraction rate [11]. Thus, MAE prevents the loss of resveratrol as it hastens the extraction time. Furthermore, this method is considered a green extraction technology because MAE requires less solvent consumption than Soxhlet extraction [12,13]. MAE has been successfully used to extract resveratrol and its derivatives from several food matrices, such as rice grain [14], grape pomace [15], and peanut skin [16]. 

To obtain the complete recovery of the targeted compound, optimization of the influencing MAE factor is needed. The efficiency of its method depends on the extraction time, extraction temperature, solvent-to-solid ratio, and microwave power [14]. In order to evaluate those parameters simultaneously, an advanced experimental design is advisable. This study aimed to optimize an MAE method for extracting resveratrol from functional marmalade and cookies by Box–Behnken design in conjunction with response surface methodology. 

## 2. Materials and Methods

### 2.1. Materials and Chemicals

HPLC-grade methanol, acetonitrile, acetic acid, and trans-resveratrol were purchased from Sigma-Aldrich (St. Louis, MO, USA). Water was purified with the Milli-Q system (Millipore Corp, Billerica, MA, USA). Commercial resveratrol Anti-Ox tablets (Acofarma, Terrassa, Spain) as a nutritional supplement were purchased from a local drugstore.

### 2.2. Functional Marmalade and Cookies Preparation

For the method development, the marmalade and cookie samples were prepared in a fully equipped in-house laboratory. The production of functional marmalade and cookies was in accordance with previous research [6]. The marmalade was prepared using a Thermomix food processor (Vorwerk, Wuppertal, Germany) for 6 min to mix milled grapes juice (250 g), sugar (250 g), and pectin (7.5 g). After homogenous, the blended ingredients were semi-pasteurized in the same instrument for 35 min at 70 °C. At the same time, the ingredient for cookies comprised grape seed (10 g), flour (45 g), oats (15 g), ground almonds (20 g), baking powder (2.5 g), oil (20 mL), salt (0.3 g), and honey (45 mL), which were blended for 20 min in a mixer (KitchenAid^®^ Artisan^®^, Mississauga, Canada). A circular mold was employed, resulting in 20 units of 7.9 g per cookie. Subsequently, the cookies were baked in an electric oven EO 2131 (Delonghi, Treviso, Italy) for 10 min at 170 °C.

To evaluate the stability of resveratrol throughout the processing conditions as an approach in the real sample application, each batch of the two functional foods (marmalade and cookies) was enriched with 210 mg of commercial resveratrol either before or after the in-house production in the laboratory. Additionally, to check the matrix effect by measuring the level of resveratrol in varied products of commercial marmalade and cookies, a spiking method was applied; thus, the recovered concentration could be higher than the limit of quantification. The spiking method was performed by grinding the sample using a homogenizer (Ultra Turrax IKA^®^ T25 Digital, Staufen, Germany) for 5 (marmalade) or 10 (cookies) min prior to the addition of resveratrol. The fortified foods were stirred until homogenous and stored in a closed container. 

### 2.3. Commercial Marmalade and Cookie Samples

For the real sample application, a number of marmalades (12 samples) and cookies (9 samples) were purchased from a local market in Cadiz, Spain. The marmalades included the brand of Tamara (Murcia, Spain) made from organic fruits (fig, peach, apricot, and strawberry), Vitalgrana (Alicante, Spain) made from organic pomegranate, and Feiner Genuss (Jacobi Scherbening GmbH & Co., KG, Padeborn, Germany) made from Morello cherry, black currant, and raspberry. While the cookie samples consisted of the following brands: Digestive Avena and Soy-orange Fontaneda (Mondelez España Commercial, S.L., Madrid, Spain), ActiSan, Tosta Rica (Cuétara S.L.U., Madrid, Spain), Maria Dorada (Cerealto Siro, Palencia, Spain), Dinosaurus (Artiach, Vizcaya, Spain), Arizona Cookies (Arizona, The Netherlands), Palmeritas (Virgen del Brezo, Palencia, Spain), GutBio (GutBio, Spain), Choco Shooters, Diet Nature Sin Azúcares, and Vanilla Wafers Sin Azúcares (Gullón, Palencia, Spain).

### 2.4. Extraction of Resveratrol

The extraction was performed by microwave-assisted extraction in a Milestone Ethos 1600 (Sorisole, Italy) using 5 g sample at 100 °C for 10 min. Afterwards, the extraction vessel was cooled down in an ice bath for 5 min. To remove the matrix effect due to the fat content in the cookies, the mixture was centrifuged (Microfriger-BL, J.P. Selecta, SA mark) at 8000 rpm (4 °C) for 10 min. The supernatant was adjusted to a volume based on the experimental design and filtered through 22 µm cellulose filters (Millipore, Merck KGaA, Darmstadt, Germany) before injection into a chromatographic system.

### 2.5. Determination of Resveratrol

The analysis was carried out using Ultrahigh Performance Liquid Chromatography (UPLC) ACQUITY UPLC^®^ H-Class system and coupled with a Fluorescence Detector (FD) controlled by Empower™ 3 Chromatography Data Software (Waters Corporation, Milford, MA, USA). The column used was C18 with a reverse phase system (ACQUITY UPLC^®^BEH C18 1.7 µm, 2.1 × 100 mm) from Waters (Ireland). The column temperature was set at 47 °C.

The chromatographic eluent contained two phases: A (0.1% acetic acid in water) and B (2% acetic acid in acetonitrile). The gradient program based on the previous research [6] was as follows (time, B%): 0 min, 0%; 1, 0%; 1.1 min,10%; 2 min, 10%; 3 min, 20%; 3.5 min, 60%; 4 min, 100%. The column was subsequently washed with 100% B for 3 min and equilibrated with 0% B for 3 min. The injection volume was 3 µL, and the wavelength was set at 310 nm for the excitation and 403 nm for the emission.

The method validation was performed following the suggestions of ISO 17025 and the recommendation in the ICH Guideline Q2 (R1) [17,18]. Hence, validation parameters covering linearity, recovery, precision, detection, and quantification limit of the method were evaluated. The linearity is significant in providing the linear responses for quantification from the tested analyte to the resveratrol concentration within the studied range. The linear curve was established using a series of dilutions from the standard resveratrol solution to cover the lower (20–100 ppb) and higher (100–700 ppb) range of concentrations. The calibration curve and regression analysis were generated using Gnumeric 1.12.17 software. (Access online through http://www.gnumeric.org/, accessed on 6 December 2022) The resulting standard deviation and the regression slope were used to calculate the limit of detection (LOD) and quantification (LOQ). The analytical characteristic for determining the content of resveratrol is presented in Table 1.

The precision of the MAE optimum condition was assessed by studying the intermediate precision (extra-day) and repeatability (intra-day). The intermediate precision was measured by individual extraction on three different days, whereas nine repetitions of extraction determined repeatability on the same day. The precision value was expressed using the coefficient of variation (CV). Based on the AOAC manual for the Peer-Verified Methods program, the accepted CV limit is ±10%. 

### 2.6. Experimental Design and Statistical Analysis

The extraction conditions must be optimized to achieve the highest recovery of the target compound. Therefore, a factorial experimental design was applicable to simultaneously evaluate three operating variables of MAE (*x*_1_: solvent composition, *x*_2_: solid-to-sample ratio, and *x*_3_: microwave power). Due to the cookies containing less water and sugar than the marmalade, evaluating and optimizing particular extraction conditions for each food matrix was necessary. The design domain for *x*_1_ was different for marmalade (60–100% methanol in water) and cookies (10–70% methanol in water), taking into account the characteristics of the matrices, specifically the low level of water in the cookie samples. On the contrary, the working range of variables *x*_2_ (5:20–5:60 *w*/*v*) and *x*_3_ (250–750 watt) was established at the same levels. 

Because each variable has different units and ranges, each of the factors was coded uniformly as −1 (low) or +1 (high) for normalization (Table 2). Meanwhile, other variables such as temperature and extraction time were kept constant at 100 °C and 10 min. Based on a previously published method by Liazid et al [19], resveratrol is stable in high-temperature exposure up to 100 °C. The levels of each variable are listed in Table 2, and the Box–Behnken design (BBD) was set for 15 experimental points (Table 3) in random order. The amount of resveratrol extracted from the studied functional food samples with varying MAE variables was used as the response.

After the BBD responses were compiled, a second-order polynomial function (Equation (1)) was established for the optimization because higher-order interactions are usually insignificant and may be confused with the main effects.

(1) $$y=\beta_{0}+\sum_{i=1}^{k}\beta_{i}x_{i}+\sum_{i=1}^{k}\beta_{ii}x_{i}^{2}+\sum_{i=1}^{k}\sum_{j=1,\;j\neq i}^{k}\beta_{ij}x_{i}x_{j}\;$$

where *x_i_*, *x_j_*,…, *x_k_* are the variables that influence the response *y*; *β*_0_, *β_ii_* (*i* = 1, 2, …, *k*), and *β_ij_* (*i* = 1, 2,…, *k*) are unknown parameters. The *β* coefficients were obtained by the partial least squares method. Statgraphics Centurion XVI (Statpoint Technologies Inc., Warrenton, VA, USA) was used to calculate the effect of the studied variables and the optimum MAE conditions.

## 3. Results and Discussion

### 3.1. Effect of MAE Variables

The influencing MAE variables to extract resveratrol from marmalade and cookies were evaluated by performing a 3-level second-order Box–Behnken design. The experiments were established as a combination of a two-level complete factorial design with an incomplete block setting that introduces the center point. As a result, experimental conditions combining all extreme levels can be omitted. The effects of solvent composition (*x*_1_), solvent-to-sample ratio (*x*_2_), and microwave power (*x*_3_) were calculated based on the analysis of variance (ANOVA), while the optimization of the MAE condition employed regression analysis that demonstrated the empirical relationship between the studied factors and the levels of resveratrol extracted from the samples. 

**Figure 1:**
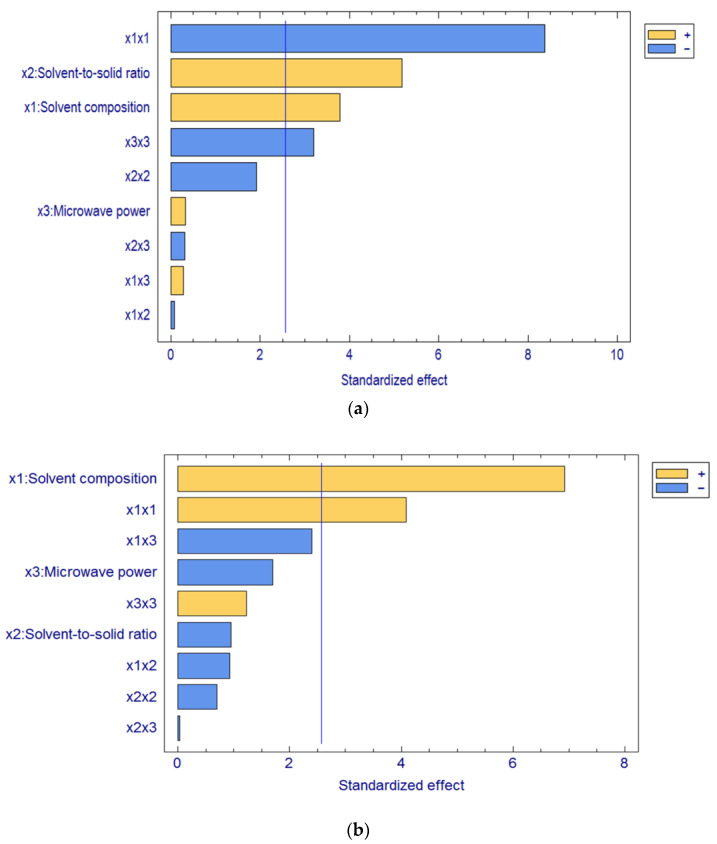
Pareto charts for the standardized effects of MAE factors in the extraction of resveratrol from (**a**) marmalade and (**b**) cookies. The significant factors are those intersected by the blue line.

The Pareto chart (Figure 1) compiles the single, interaction, and quadratic effects of MAE factors in decreasing order of importance. The significant effects with a *p*-value lower than 0.05 are represented by bars crossing the vertical line in the Pareto chart. Hence, the most influencing factor was the solvent composition (*x*_1_) for resveratrol extraction from marmalade and cookies, whilst microwave power (*x*_3_) did not affect the extraction of resveratrol from both samples. In contrast, the solvent-to-solid ratio (*x*_2_) only significantly affected the resveratrol extraction from the marmalade. Additionally, there was no significant effect due to the interaction between MAE factors but quadratic effects of solvent composition (*x*_1_*x*_1_) in both samples and microwave power (*x*_3_*x*_3_) in marmalade. 

The estimated coefficients were subsequently calculated to build a predictive model for the level of resveratrol extracted from both samples using only the significant factors. It showed by the factors which intersected by the blue line. The equation of fitted models of marmalade (Equation (2)) and cookies (Equation (3)) are as follows:

(2) $$y=2.209+0.210x_{1}+0.286x_{2}–0.682\;x_{1}x_{1}–0.260x_{3}x_{3}$$

(3) $$y=0.128+1.736x_{1}+1.509x_{1}x_{1}$$

The effectiveness and applicability of the models were confirmed by considering the high coefficient of determination (*R*^2^) from regression statistics (95.98 and 93.91% for marmalade and cookies, respectively). Therefore, the models provided prediction values that fitted with the experimental results (mean absolute error were 0.073 and 0.323 for marmalade and cookies, respectively). 

### 3.2. Optimization of Influencing Factors

**Figure 2:**
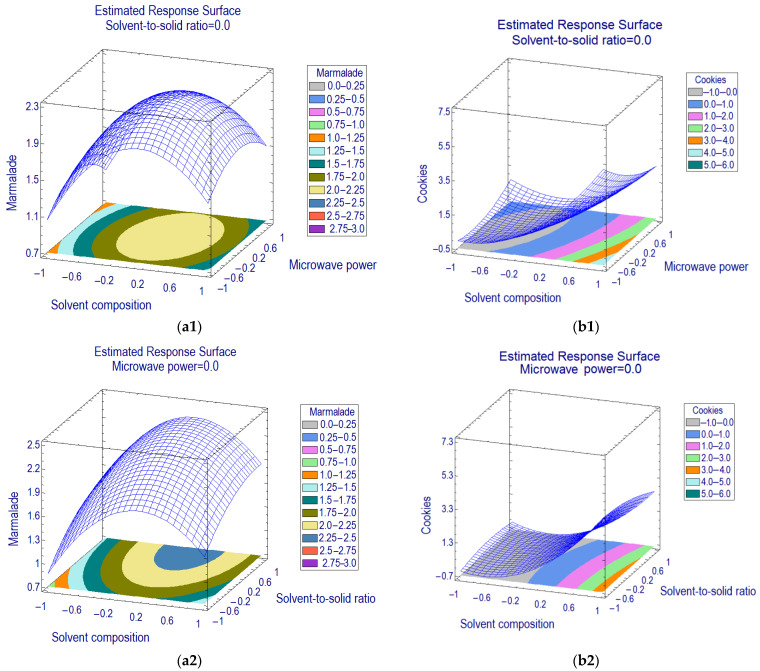
Response surface and contour plots displaying effects of MAE factors on the level of extracted resveratrol from (**a**) marmalade and (**b**) cookies.

The three-dimensional response surface methodology (RSM) on the basis of a proposed second-order polynomial model has been plotted (Figure 2) by assigning the level of resveratrol as the response in the *y*-axis against two MAE factors in the *x* and *z*-axes, whilst the level of another independent factor was fixed at the center point.

The common influencing factor of MAE for marmalade and cookie matrices was the solvent composition (*x*_1_). A mixture of methanol–water is suitable for extraction in a microwave system due to its dielectric constant and dielectric loss [20]. Furthermore, a mixture of methanol–water is proven to efficiently extract the resveratrol from several food matrices [6,21]. The optimization of solvent composition can be achieved by increasing the percentage of methanol in water (estimated effect +3.47 and +0.42 for marmalade and cookies, respectively). 

**Figure 3:**
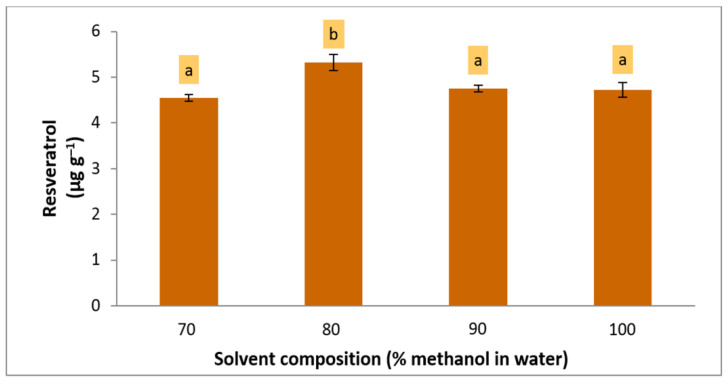
Further optimization for solvent composition in the extraction of resveratrol from cookie samples. Different letters in the bars mean significant differences (95% confidence level).

Since the optimum coordinate of solvent concentration for cookie samples was near the corner of the design domain (+0.995, 70%), a higher percentage of methanol than the range used in the experimental design (70, 80, 90, and 100%) was included in the assessment before maximizing the recovery of the extraction method. The level of resveratrol was increased when the concentration of methanol in water increased. However, 80% methanol in water significantly provided the highest resveratrol recovery (LSD, *p* = 0.05) (Figure 3). In contrast, the optimum coordinate for solvent composition for marmalade was within the design range (+0.149); thus, 79% methanol in water was defined as the optimum extraction solvent.

In addition to solvent composition, the solvent-to-solid ratio (*x*_2_) positively affected the extraction (estimated effect +0.572) of resveratrol from marmalade samples. Therefore, a higher ratio of solvent-to-sample provided an advantage of higher mass transfer of resveratrol in the sample matrix into solvents. Conversely, the estimated effect of microwave power (*x*_3_) was nonsignificant, but the quadratic effect (*x*_3_*x*_3_) was (estimated effect −0.520). Hence the second-order model suggested the optimum condition for extracting the resveratrol from marmalade was set in the center level of microwave power (500 W), solvent-to-sample ratio (50:4), and solvent composition (80% methanol in water). In comparison, the optimum condition for extracting resveratrol from cookie matrices using MAE was applying 20:5 for setting the solvent-to-sample ratio, 250 W for microwave power, and 80% methanol in water for the extraction solvent.

### 3.3. Extraction Kinetics

**Figure 4:**
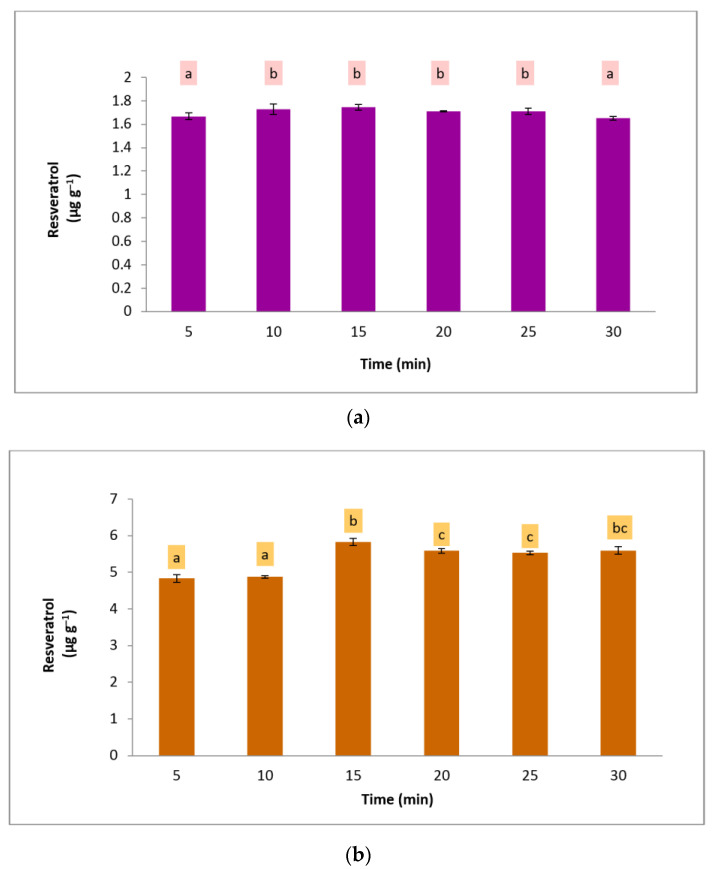
The optimized MAE condition in different extraction times for extracting resveratrol from (**a**) marmalade and (**b**) cookie samples. Different letters in the bars mean significant differences (95% confidence level).

In order to determine the optimum extraction time for the targeted analyte recovery, extraction kinetics was studied. The recovery of the resveratrol was measured every 5 min intervals within 5 to 30 min of extraction. Figure 4 presents the recovery of each extraction time. For both functional foods, 15 min extraction produced the highest resveratrol levels. For cookie matrices, 15 min and 30 min were not significantly different (*p* < 0.05); however, 15 min was chosen due to the energy saving of the process. While, for the marmalade sample, the resveratrol recovery after 10 min was not significantly different (*p* < 0.05) up to 25 min. Finally, 15 min was set as the optimum extraction time because it clearly showed a lower standard deviation than 10 min. 

Overexposure to MAE may cause the loss of some phenolic compounds [22,23,24]. An extended extraction time for both functional food samples reduced the extracted resveratrol. It was relevant to the previous finding that a longer extraction time might cause undesirable reactions, such as enzymatic degradation and oxidation, which decrease the recovery of phenolic compounds [25]. Thus, a shorter MAE time is favorable; apart from avoiding the degradation of the compounds, it is also more energy-saving than conventional extraction techniques [25]. 

### 3.4. Precision of MAE Method

The optimum conditions of the developed MAE to recover resveratrol from marmalade and cookie samples (Table 4) were then validated in terms of precision, according to the ICH guideline. The precision was measured as repeatability and intermediate precision and expressed as coefficient of variation (CV). The precision values of both MAE methods were lower than 5.70% for repeatability (*n* = 9) and 5.34% for intermediate precision (*n* = 3 × 3). The CV values were satisfactory and below the acceptable limit (±10%), indicating that the developed MAE methods have high precision for extracting resveratrol from marmalade and cookies.

### 3.5. Matrix Effect on Resveratrol Recoveries

**Figure 5:**
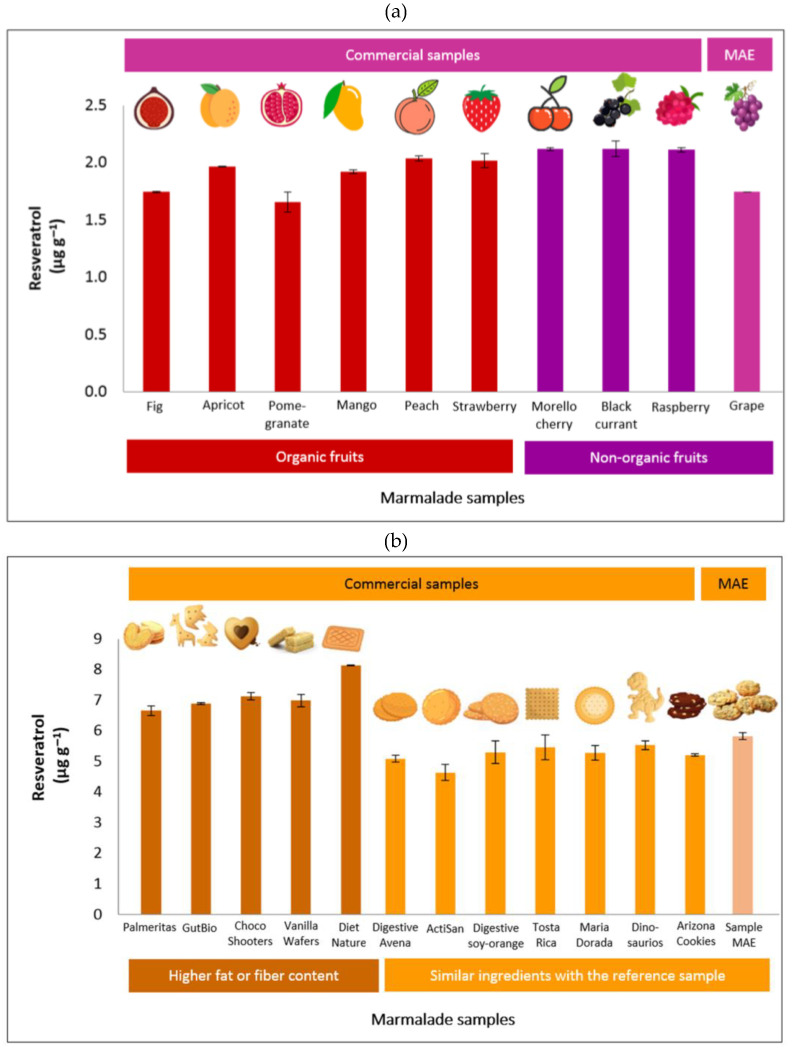
Quantification of resveratrol in commercial products (**a**) marmalade and (**b**) cookies.

The optimized and valid method was used to extract a large number of samples to check for the matrix effects. This approach determined the quantity of resveratrol in several brands and types of marmalade and cookie products available in the market. For marmalade samples, nine commercial products from organic (fig, apricot, pomegranate, mango, peach, strawberry) and non-organic (Morello cherry, black currant, and raspberry) fruits were used. It was found that the commercial marmalade samples did not contain resveratrol (LOD = 1.5 ppb). Hence, the spiking technique was applied to study the accuracy of MAE for determining resveratrol in marmalade samples. The same level of resveratrol added to the marmalade sample for developing the MAE method was also spiked in all studied marmalade samples. The resulting recoveries are shown in Figure 5. 

The recoveries of resveratrol in marmalade samples prepared from organic fig and pomegranate were lower than the reference (marmalade sample prepared during the MAE method development). In contrast, the other seven commercial marmalades contain a higher amount of resveratrol recovered using MAE compared with the reference sample. By comparing the recovery values, the difference was less than 0.4 μg g^−1^ between the commercial and reference samples. Hence, the optimized method effectively extracted the resveratrol from all studied marmalade samples.

A similar approach to assess the matrix effect was also applied to cookie samples. Twelve commercial cookies from the local Spanish market were evaluated, including Digestive Avena, ActiSan, Digestive soy-orange, Tosta Rica, Maria Dorada, Dinosaurus, Arizona Cookies, Palmeritas, GutBio, Choco Shooters, Diet Nature, and Vanilla Wafers. The first seven cookies provided comparable characteristics to the reference (cookie sample prepared during the MAE method development) as they were prepared using similar ingredients. In contrast, the last five samples have specific characteristics: Palmeritas brand contains high sugar and margarine; GutBio contains butter, cocoa powder, and beet molasses; Choco Shooters is chocolate-based cookies filled with heavy cream; vanilla wafers made of 70% cream, traces of nuts and milk, and; Diet Nature provide a high dietary fiber from vegetable and pea.

In all those commercial sample cookies, the resveratrol compound was not detected as the response was lower than the detection limit; thus, the spiking technique was also applied to check the recovery ability of the MAE method. Figure 5 confirms that the proposed MAE method was suitable for the first seven samples due to similar matrices to those in the study. While the high-fat cookies and those that contained high fiber resulted in overestimated values up to 2.3 µg g^−1^ because the matrices included interfering ingredients. 

### 3.6. MAE Application

The method was also used to measure the degradation of resveratrol during the making process of marmalade and cookie samples. Due to exposure to a semi-pasteurization treatment (70 °C, 35 min) to produce marmalade from grape juice, the level of resveratrol was decreased by 12.4%. In contrast, the heating process for cookie samples from grape seed employed an oven temperature of 170 °C for 10 min resulting in resveratrol degradation by 14%. Henceforth, it is interesting to conclude that the inclusion of resveratrol is feasible without worrying about degradation during the preparation of these foods. 

The approach described here ensures the stability of resveratrol that is naturally present in wine by-products and added as an ingredient to enrich functional foods. This aspect is relevant for developing functional foods that include the use of by-products from the wine industry.

## 4. Conclusions

Two analytical methods for microwave-assisted extraction (MAE) to determine resveratrol in marmalade and cookie samples have been successfully developed and validated. The most influencing extraction factor was the solvent composition, while the solvent-to-sample ratio only affected the resveratrol extraction from the marmalade. The optimum conditions of the studied factors to extract resveratrol from marmalade (microwave power, 500 W; solvent-to-sample ratio, 50:4; and solvent composition, 80% methanol) and cookie matrices (microwave power, 250 W; solvent-to-sample ratio, 20:5; and solvent composition 80% methanol) were successfully defined. Kinetic studies confirmed 15 min as the suitable extraction time for a complete resveratrol recovery applying the optimum MAE condition for marmalade (1.74 µg g^−1^) and cookie (5.53 µg g^−1^) samples. The developed MAE was validated with high precision and applicability for some commercial marmalade and cookie products available in the market. Additionally, MAE was used to measure resveratrol degradation due to the heating process while preparing grape-derived products as functional marmalade and cookies. The maximum degradation of resveratrol in both foods was not higher than 15% despite being very sensitive to light and high temperatures. Hence, the MAE can be used as an analytical tool to assist in developing functional foods.

## Figures and Tables

**Table 1:** The analytical properties of the chromatographic method for the determination of resveratrol using UHPLC-FD.

Linear Range (ppb)	Linear Equation	*R* ^2^	LOD (ppb)	LOQ (ppb)
10−100	*y* = 181.82*x* + 155	0.998	12	37
100−700	*y* = 288.67*x* + 13236	0.997		

**Table 2:** Coding table of experimental factors and levels in microwave-assisted extraction.

MAE Factors		Levels			Unit
		−1	0	+1	
*x*_1_, Solvent composition	Marmalade	60	80	100	% methanol in water
	Cookies	10	40	70	% methanol in water
*x*_2_, Solvent-to-solid ratio		20:5	40:5	60:5	mL solvent g sample^−1^
*x*_3_, Microwave power		250	500	750	Watt

**Table 3:** Experimental factors of Box–Behnken design and the observed level of resveratrol extracted from marmalade and cookies (mean ± SD) by MAE.

Run	*x*_1_ Solvent Composition	*x*_2_ Solvent-to-Solid Ratio	*x*_3_ Microwave Power	Resveratrol (µg g^−1^)	
				Marmalade	Cookies
1	0	0	0	2.188 ± 0.011	0.127 ± 0.008
2	0	−1	1	1.678 ± 0.052	0.485 ± 0.042
3	−1	0	1	1.035 ± 0.013	0.000 ± 0.000
4	0	0	0	2.245 ± 0.113	0.143 ± 0.022
5	0	0	0	2.193 ± 0.039	0.119 ± 0.013
6	0	−1	−1	1.410 ± 0.064	0.468 ± 0.006
7	−1	0	−1	1.225 ± 0.022	0.001 ± 0.000
8	−1	−1	0	0.759 ± 0.035	0.000 ± 0.000
9	−1	1	0	1.418 ± 0.020	0.002 ± 0.002
10	1	0	1	1.351 ± 0.079	2.485 ± 0.059
11	0	1	−1	1.958 ± 0.079	0.191 ± 0.032
12	1	−1	0	1.337 ± 0.019	3.423 ± 0.178
13	0	1	1	2.127 ± 0.002	0.165 ± 0.027
14	1	1	0	1.970 ± 0.169	2.100 ± 0.005
15	1	0	−1	1.456 ± 0.069	5.884 ± 0.069

**Table 4:** Optimized MAE conditions to extract resveratrol from marmalade and cookie samples.

Samples	Solvent Composition (%Methanol)	Solvent-to-Solid Ratio (mg g^−1^)	Microwave Power (W)	Extraction Time (min)
Marmalade	80	50:4	500	15
Cookie	80	20:5	250	15

## Data Availability

Data is contained within the article.
